# Expiration-Triggered Sinus Arrhythmia Predicts Mortality Risk in the General Elderly Population

**DOI:** 10.3390/jcdd12020040

**Published:** 2025-01-24

**Authors:** Ralf Josef Dirschinger, Alexander Müller, Alexander Steger, Karl-Ludwig Laugwitz, Petra Barthel, Georg Schmidt, Daniel Sinnecker

**Affiliations:** 1 Department of Internal Medicine I, University Hospital rechts der Isar, TUM School of Medicine and Health, Technical University of Munich, 81675 Munich, Germany; 2 Gefäßpraxis im Tal, 80331 Munich, Germany; 3 Department Clinical Medicine, TUM School of Medicine and Health, Technical University of Munich, 81675 Munich, Germany; 4 MVZ Harz, 38640 Goslar, Germany

**Keywords:** risk stratification, respiratory sinus arrhythmia, cardiac autonomic function, heart rate variability, phase-rectified signal averaging

## Abstract

Reduced respiratory sinus arrhythmia, quantified as expiration-triggered sinus arrhythmia (ETA) from simultaneous electrocardiogram and respiration recordings, is a strong long-term mortality predictor in myocardial infarction survivors. Here, we investigated whether ETA also predicts mortality risk in the general elderly population. ETA was quantified from 30-min electrocardiogram and respiration recordings in 1788 general population subjects aged ≥60 years, who were then followed for a median of 4.0 years (median age 72 years, 58% female). Four-year all-cause mortality was 4.6%. Abnormal ETA using a predefined cutoff (≤0.19 ms) was associated with a 4-year mortality of 6.9%, compared to 3.7% in the remaining participants (*p* = 0.0022). ETA remained a significant mortality predictor in multivariable Cox analysis, also considering a modified Framingham score incorporating sex, age, smoking, cholesterol, blood pressure, antihypertensive medication, family history, diabetes and clinical atherosclerosis (multivariable hazard ratio 1.81; 95% confidence interval 1.17–2.81; *p* = 0.008). Combined risk prediction by ETA (using an optimized cutoff of ≤0.86 ms) and the Framingham score stratified patients into a low-risk (both parameters normal), an intermediate-risk (one parameter abnormal) and a high-risk group (both parameters abnormal), with 4-year mortality rates of 1.9%, 4.4% and 10.1%, respectively. We conclude that in elderly general population subjects, ETA is a mortality risk predictor that complements classical clinical risk stratification.

## 1. Introduction

As the global population ages, it becomes increasingly important for public health and clinical practice to understand the predictors of mortality in elderly individuals. Cardiovascular health is a vital aspect of overall well-being and a major determinant of mortality in older adults, and heart rate variability (HRV) has been long recognized as an indicator of cardiac autonomic function and, consequently, a predictor of mortality risk [1]. One specific measure of HRV, respiratory sinus arrhythmia (RSA), reflects the interaction between the respiratory and cardiovascular systems, which is mediated by the autonomic nervous system. It provides insights into cardiac vagal tone [2,3,4].

Expiration-triggered sinus arrhythmia (ETA), a refined measure of RSA calculated by bivariate phase-rectified signal averaging [5] from simultaneous electrocardiogram (ECG) and respiration recordings to quantify the amount of heart rate slowing associated with expiration, has garnered attention as a strong and independent predictor of long-term mortality in survivors of acute myocardial infarction [6]. However, given that this parameter has so far only been investigated in one cohort of post-infarction patients, it was unclear whether its predictive power could be validated in different cohorts or in other clinical settings [7].

The present cohort study aimed to investigate the relationship between ETA and mortality in a diverse group of elderly individuals from the general population with and without pre-existing cardiovascular disease. By employing electrocardiographic and respiratory recordings, we measured ETA and followed participants over a four-year period for all-cause mortality. Our objective was to determine whether ETA can also serve as a predictor of mortality in this population, hypothesizing that lower levels of ETA will be associated with increased mortality risk in elderly individuals from the general population.

## 2. Materials and Methods

This study is a retrospective analysis of data from the previously described INVADE (Intervention Project on Cerebrovascular Disease and Dementia in the District of Ebersberg) study [8,9], which is a population-based study carried out in the primary care setting in the catchment area of Ebersberg in Upper Bavaria, Germany. Between August 2013 and February 2015, all residents of the area aged ≥60 years who were insured by the health insurance company Allgemeine Ortskrankenkasse (AOK) Bayern, Munich, Germany, the largest Bavarian health insurance firm, were invited to participate. The study complied with the Declaration of Helsinki, was approved by the local ethics committee, and all study participants gave written informed consent.

All participants underwent a baseline visit during which simultaneous non-invasive 30 min recordings of ECG (5-electrode ECG sampled at 300 Hz) and respiration by chest impedance were obtained in a supine resting position in a quiet and undisturbed environment. ECG signal analysis consisted of automated artefact elimination and QRS complex morphology classification followed by manual review by experienced technicians blinded to clinical outcome data. ETA was calculated by bivariate phase-rectified signal averaging, as described previously [6]. Briefly, using custom-written code in Matlab (The MathWorks, Inc., Natick, MA, USA), the respiration signal was converted into an analytical signal ranging from −1 (beginning of inspiration) to +1 (end of inspiration). ECG segments surrounding heartbeats lying within the expiratory phase (defined by predefined thresholds ϕ1 (0.6) and ϕ2 (−0.1) [6]) were averaged after aligning them on these anchor heart beats, and ETA was calculated by Haar wavelet analysis from the averaged signal as the expiration-related change in RR intervals. Instead of the chest belt-derived respiration signal used in the initial description of the method, we used the impedance-based signal without any changes in the parameters or the algorithm, particularly the ϕ1 and ϕ2 threshold values defining the expiration phase [6]. For the analysis presented here, only participants who had sinus rhythm during the baseline visit, in whom the quality of ECG and respiration signals allowed the quantification of ETA, were included, resulting in a total of 1788 study participants. Heart rate turbulence (HRT) [10] and deceleration capacity of heat rate (DC) [11] were determined from the ECG recording as described previously [9].

A modified Framingham score, which was calibrated to the German setting and is widely used among German general practitioners (http://www.arriba-hausarzt.de (accessed on 1 December 2024)), was calculated for each study participant [12] based on age, sex, current smoking status, total cholesterol, high-density lipoprotein (HDL) cholesterol, systolic blood pressure, antihypertensive medication use, family history of cardiovascular disease, clinical evidence of diabetes mellitus with hemoglobin (Hb)A1 c in diabetic patients, and clinical evidence of atherosclerosis. The score is expressed as the expected 10-year risk for cardiovascular events or death in percent. As described previously [9], we prospectively chose score values of ≥20% and <20% to define high- and low-risk groups, respectively. Patients were considered to be taking antihypertensive medication if their medication included any drug of the substance classes beta-blockers, calcium antagonists, angiotensin-converting enzyme (ACE) inhibitors, angiotensin (AT)1 inhibitors or diuretics. Patients were considered as having manifest cardiovascular disease if they had a history of acute myocardial infarction, coronary artery disease, peripheral artery disease or major stroke.

The participants were followed over a median period of 4.0 years (interquartile range 3.6–4.3 years). The prospectively defined primary end point was all-cause mortality. Participants who did not adhere to the scheduled follow-up were contacted by letter or by telephone; if this was not successful, the local population register was used to ascertain the survival status of the participants. There were no participants lost to follow-up. Upon study termination in February 2018, all participants had reached either the primary end point or the prospectively defined minimum follow-up period of 30 months.

The distribution of quantitative data is presented as median and interquartile range. Qualitative data are described as absolute and relative frequencies. For an exploratory analysis, time-dependent receiver-operating characteristic (ROC) curve analysis [13] was performed and the optimized dichotomy for ETA in this cohort was determined as the maximum Youden’s J index (sensitivity + specificity −1) [14]. Group differences between Kaplan–Meier estimates were assessed by log-rank tests. Univariable and multivariable Cox proportional hazard regression models were used to assess hazard ratios (reported with 95% confidence intervals (95% CI)) of risk predictors. Analyses were performed using R [15] version 4.4.2.

## 3. Results

The baseline characteristics of the study population are shown in Table 1. Cardiovascular risk factors such as hypertension, hyperlipidaemia, diabetes mellitus or smoking were present in a vast majority of the study subjects, who had a median modified Framingham score (estimating the 10-year risk of major cardiovascular events or death) of 14.4%. Manifest cardiovascular disease was present in roughly a quarter of the study subjects. Over a median follow-up of 4 years, 82 study subjects died, amounting to an all-cause mortality of 4.6% (see Table 1). Compared to the study subjects who were still alive at the end of follow-up, the non-survivors were older, more often male, more often current smokers, had a higher modified Framingham score, and a higher prevalence of manifest cardiovascular disease and renal insufficiency (see Table 1).

**Figure 1:**
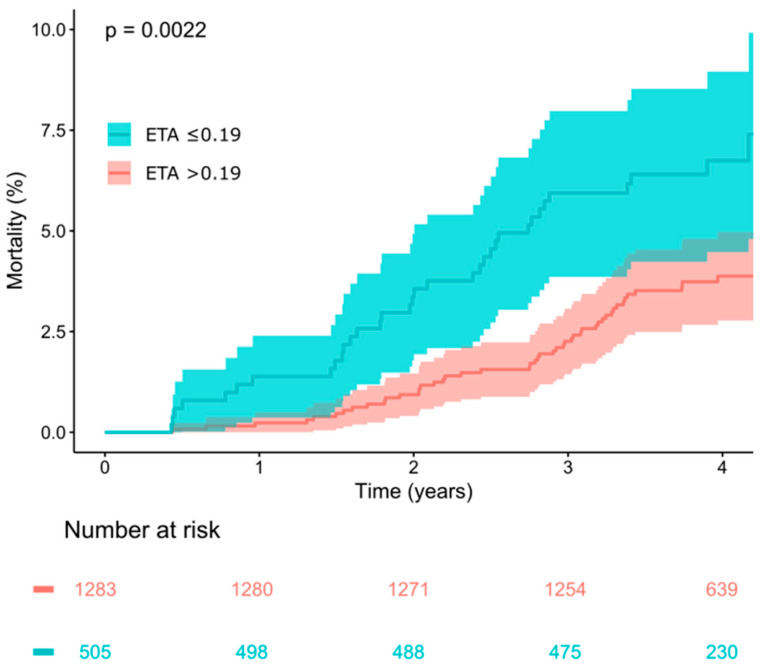
Survival in study participants stratified according to expiration-triggered sinus arrhythmia (ETA) using the predefined dichotomy of ≤0.19 ms. Kaplan–Meier curves along with 95% confidence intervals are shown. The numbers of patients at risk in the respective groups are shown below the graph.

The parameter ETA was developed as a risk predictor in a cohort of survivors of acute myocardial infarction [6]. In the derivation cohort, the optimum dichotomy to predict all-cause mortality was ETA ≤ 0.19 ms. In the elderly general population studied here, 505 of the 1788 study subjects (28.2%) had a blunted respiratory sinus arrhythmia signified by ETA values ≤ 0.19 ms. In these subjects, all-cause mortality during follow-up was 6.9% (35/505), in contrast to a significantly lower mortality rate of 3.7% (47/1283) in the subjects with more pronounced respiratory sinus arrhythmia, indicated by ETA values > 0.19 ms. Kaplan–Meier survival curves of subjects dichotomized by ETA are shown in Figure 1.

In univariable Cox analysis, ETA ≤ 0.19 ms was a significant predictor of all-cause mortality with a hazard ratio of 1.96 (see Table 2). The association of ETA ≤ 0.19 ms with mortality remained significant in multivariable analysis that also considered the modified Framingham score, which takes into account sex and age as well as smoking status, total and HDL cholesterol, blood pressure, antihypertensive medication use, family history of cardiovascular disease, diabetes mellitus and clinical evidence of atherosclerosis. The multivariable hazard ratio for ETA ≤ 0.19 ms was 1.81 (see Table 2). The association was also present in univariable and multivariable Cox analysis considering ETA as a continuous variable (see Table 3. Note that reduced respiratory sinus arrhythmia, indicated by reduced ETA, is associated with increased mortality, resulting in hazard ratios < 1 for ETA as a continuous parameter).

ETA was also independent of other HRV-based autonomic parameters such as heart rate turbulence (HRT) and deceleration capacity of heat rate (DC). When ETA (dichotomized at ≤0.19 ms) was entered into a multivariable Cox model for all-cause mortality, together with the HRT turbulence slope (dichotomized at ≤2.5 ms) and DC (dichotomized at ≤2.5 ms), abnormal ETA remained significantly associated with all-cause mortality, with a hazard ratio of 1.65 (95% CI 1.04–2.62, *p* = 0.034).

**Figure 2:**
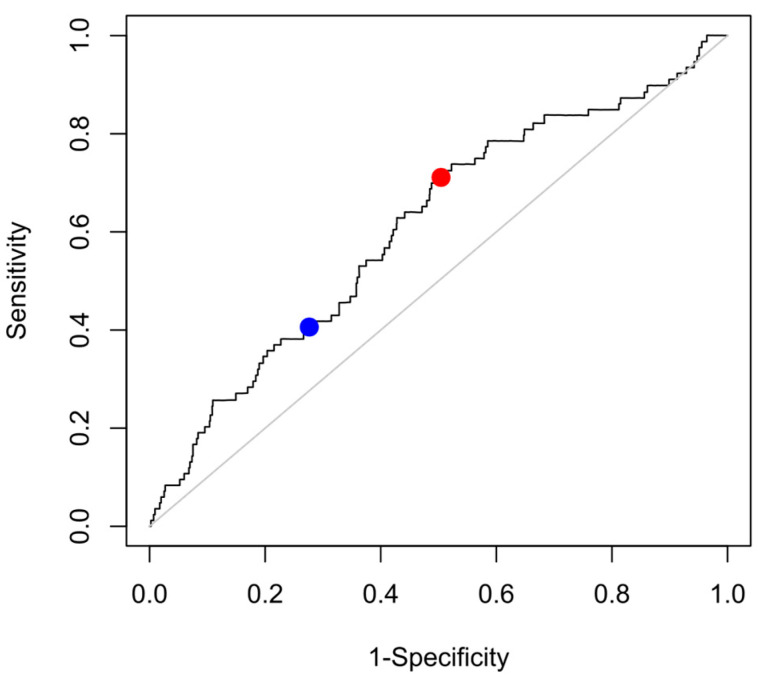
Receiver-operating characteristic (ROC) analysis for the prediction of four-year all-cause mortality by ETA. The points on the ROC curve corresponding to the predefined (≤0.19 ms) and new (≤0.81) dichotomies are highlighted in blue and red, respectively. The line of identity is shown in grey.

**Figure 3:**
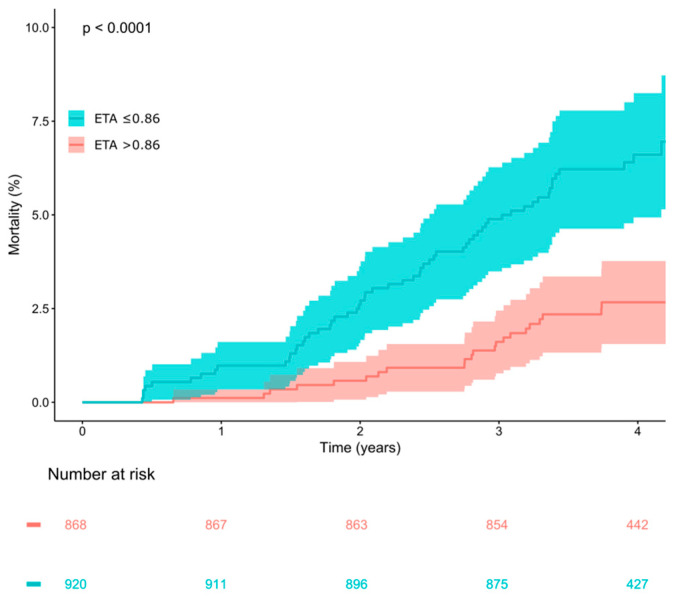
Survival in study participants stratified according to expiration-triggered sinus arrhythmia (ETA) using the optimized dichotomy of ≤0.86 ms. Kaplan–Meier curves along with 95% confidence intervals are shown. The numbers of patients at risk in the respective groups are shown below the graph.

The cohort of survivors of acute myocardial infarction in whom the parameter ETA was developed had a considerably higher mortality rate than the elderly subjects from the general population studied here (their five-year mortality rate was 7.7% [6], compared to a mortality of 4.6% over the median follow-up duration of 4.0 years in this study). The median ETA value in the post-infarction cohort was 0.54 ms (IQR −0.25–1.66 ms) [6], which is markedly lower (i.e., more pathologic) than the 0.81 (IQR 0.10–1.81) measured in the participants of this study, indicating that the elderly subjects from the general population studied here had overall more pronounced respiratory sinus arrhythmia, indicating more functional vagal control of the heart rate as compared to the post-infarction patients. We therefore wondered whether a different dichotomy than the predefined cut-point of ETA ≤ 0.19 ms, which was optimized to discern high- and low-risk post-infarction patients, might be better suited to risk-stratify the elderly subjects from the general population studied here. The optimal dichotomy in this cohort based on receiver-operating characteristic analysis and Youden’s J index was ETA ≤ 0.86 ms (Figure 2). Of the 1788 study subjects, 920 (51.5%) had ETA values ≤ 0.86 ms, while the remaining 868 (48.5%) had ETA values > 0.86 ms. We tested the performance of ETA using this dichotomy as an exploratory analysis. Kaplan–Meier curves for subjects stratified by ETA using this new dichotomy are shown in Figure 3, indicating an even better discrimination between high-and low-risk subjects as compared to the original dichotomy of ETA ≤ 0.19 (compare to Figure 1). All-cause mortality in groups with ETA ≤ 0.86 and >0.86 ms amounted to 60/950 (6.32%) and 22/868 (2.52%), respectively (*p* < 0.0001).

**Figure 4:**
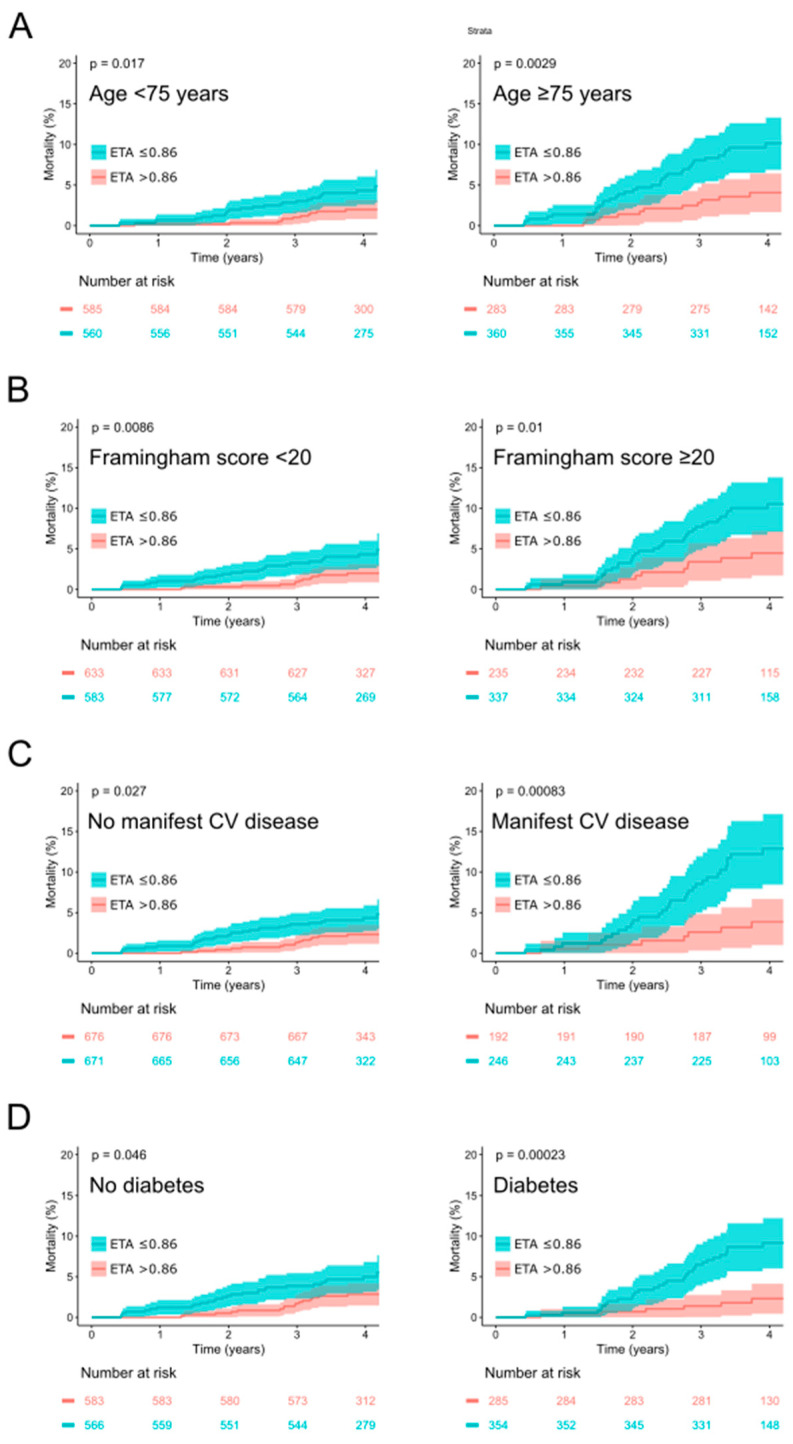
Survival in study participants stratified according to expiration-triggered sinus arrhythmia (ETA) using the optimized dichotomy of ≤0.86 ms in subgroups defined by age (**A**), the modified Framingham score (**B**), the presence of manifest cardiovascular disease (**C**) or the presence of diabetes mellitus (**D**). Kaplan–Meier curves along with 95% confidence intervals are shown. The numbers of patients at risk in the respective groups are shown below the graph.

To investigate whether the prognostic utility of ETA depends on the presence of other risk factors, we analyzed its performance as a mortality predictor in subgroups stratified according to age (≥75 years vs. <75 years), the modified Framingham score (predicted risk ≥ 20% vs. <20%), the presence of manifest cardiovascular disease and the presence of diabetes mellitus (see Figure 4).

In all the investigated subgroups, ETA ≤ 0.86 ms was associated with a significantly increased mortality rate (albeit reaching only borderline significance (*p* = 0.046) in the subgroup of study participants without diabetes mellitus (see Figure 4D)). Notably, the mortality difference between high-risk and low-risk groups stratified by ETA was always more pronounced in the subgroups with the higher overall mortality rate, i.e., in subjects of ≥75 years of age, with a modified Framingham score of ≥20%, with manifest cardiovascular disease and with diabetes mellitus (see Figure 4).

**Figure 5:**
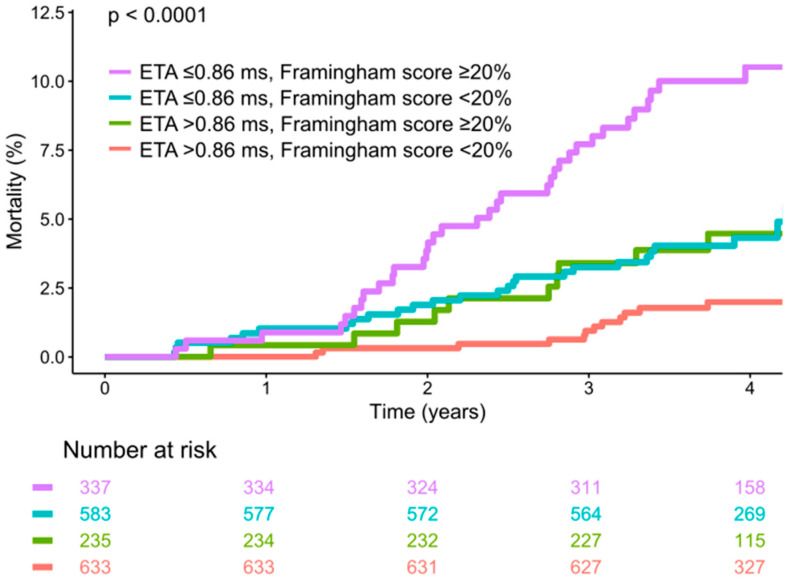
Survival in study participants stratified by expiration-triggered sinus arrhythmia (ETA) and the modified Framingham score. Kaplan–Meier curves along with 95% confidence intervals are shown. The numbers of patients at risk in the respective groups are shown below the graph.

Figure 5 shows mortality curves of study subjects stratified by the combination of both ETA (dichotomized at ≤0.86 ms) and the modified Framingham score (dichotomized at ≥20%).

The study participants for whom both ETA and the modified Framingham score indicated a low risk (ETA > 0.86 ms, Framingham score < 20%) constituted the largest group of 633/1788 subjects (35.4%), which was characterized by a low mortality rate of 12/633 (1.9%). The 235 participants (13.1%) in whom ETA indicated a low risk while the Framingham score indicated a high risk (ETA > 0.68 ms, Framingham score ≥ 20%) had an intermediate mortality rate of 4.3% (10/235), very similar to the 583 participants (32.6%) with high-risk ETA values and a low-risk Framingham scores (ETA ≤ 0.86, Framingham score < 20%), whose mortality rate was 4.5% (26/583). Thus, for risk stratification purposes, these two groups with virtual identical mortality can be combined into a large group (46% of the study population) with an intermediate mortality rate of 4.4% (36/818). In 18.8% (337/1788) of the study participants, both ETA and the Framingham score predicted a high risk (ETA ≤ 0.86, Framingham score ≥ 20%). These study subjects were characterized by a substantial mortality of 10.1% (34/337) over the median follow-up of 4 years (see Figure 5).

## 4. Discussion

The findings of this cohort study provide compelling evidence that expiration-triggered sinus arrhythmia (ETA) is an independent and significant predictor of mortality in elderly individuals from the general population. Our results show that abnormal ETA, defined using the predefined cutoff of ≤0.19 ms, was associated with a significantly higher 4-year mortality rate compared to individuals with normal ETA (6.9% vs. 3.7%, *p* = 0.0022). Furthermore, ETA remained a robust predictor of mortality even after adjusting for established cardiovascular risk factors in multivariable Cox regression analysis. This underscores the potential of ETA as a useful non-invasive tool for assessing mortality risk, particularly in the context of aging populations where conventional risk factors may only explain part of the variability in long-term outcomes. The suitability of ETA as a component of a multi-parameter polyscore to predict cardiovascular outcomes has been demonstrated previously [9,16,17].

In multivariable analysis, ETA demonstrated a hazard ratio of 1.81 (*p* = 0.008), suggesting that a decrease in ETA reflects an increased risk of mortality, independent of the risk prediction provided by the modified Framingham score which incorporates conventional risk predictors such as age, sex, smoking status, blood pressure, cholesterol levels, and clinical atherosclerosis. This supports the hypothesis that ETA, as a marker of autonomic function, provides additional risk information beyond traditional risk factors.

As an exploratory analysis, we investigated whether a different dichotomy than that developed in the post-infarction cohort [6] might even improve risk stratification in this cohort of elderly subjects from the general population. We found that improved discrimination between low- and high-risk individuals can be achieved by not using the original dichotomy (ETA ≤ 0.19 ms) but a dichotomy optimized in this population, classifying individuals as high-risk at ETA ≤ 0.86 ms (i.e., at less abnormal values than in the original dichotomy).

It is not surprising that a risk stratification parameter that was developed in a high-risk population (five-year all-cause mortality was 7.7% in that cohort of myocardial infarction survivors) shows suboptimal performance if it is transferred to a population with a lower overall risk using the identical dichotomy. The ETA values of survivors and non-survivors exhibit a substantial overlap, and there is no a priori way to define “normal” and “abnormal” values. If the original dichotomy (ETA ≤ 0.19 ms) is used in the INVADE cohort, only 28% of the subjects show “abnormal” ETA values. With the optimized dichotomy (ETA ≤ 0.86 ms), this number increases to 51%, which increases the sensitivity of the test at the expense of some specificity (see Figure 2). Using this cutoff, ETA works particularly well within high-risk subgroups (see Figure 4) and in conjunction with the modified Framingham score (see Figure 5). These findings suggest that incorporating ETA into existing clinical risk prediction models could enhance the accuracy of mortality risk assessment, particularly in the elderly, who often exhibit a complex interplay of multiple risk factors. However, the optimization of the cutpoint for ETA in the cohort under study may have resulted in over-fitting to this particular data set, possibly leading to an over-optimistic estimate of the predictive power of this approach. Therefore, the results of this exploratory subanalysis should be regarded as hypothesis-generating until validated in independent cohorts, which will be essential to confirm the broader applicability of this threshold.

One of the key strengths of this study is its focus on a cohort from the general population, which underscores the potential utility of ETA as a simple, non-invasive measure that can be easily integrated into routine clinical practice. While such an implementation may involve challenges such as the cost of the equipment, the training of the staff involved, and the time investment for patients and healthcare personnel, these should be nonetheless manageable. Given that ETA can be derived from standard ECG and respiratory measurements, its implementation would not require specialized equipment or invasive procedures, making it a practical tool for widespread use in both research and clinical settings. Whether meaningful data can also be obtained by using shorter recording periods than the 30 min used in this study, which would make implementing the technology into everyday medicine even more feasible, remains to be investigated.

We do not have serial measurements of ETA in the same subjects and thus cannot make a statement on the reproducibility of ETA measurements.

While the incorporation of the modified Framingham score into our analysis results in adjustment for a broad range of clinical risk factors, residual confounding factors cannot be fully excluded, particularly given the potential influence of factors such as ejection fraction, B-type natriuretic peptide levels, physical activity, frailty or unknown underlying non-cardiac comorbidities that were not systematically assessed in this study. This study was limited to a small region in Germany, which may limit the global applicability of the findings, especially to populations of a different ethnicity.

Based on the original description of ETA in survivors of acute myocardial infarction [6], it has been hypothesized that reduced ETA might identify patients at increased risk of sudden cardiac death due to arrhythmia [7]. The data from the present study are insufficient to support such a link between reduced ETA and arrhythmic death in the general population. Of the 82 deaths that occurred in the study population during follow-up, only 15 (18%) could be clearly attributed to a cardiac cause, and there was no clear and significant association between ETA and cardiac mortality. While this might very well just reflect a lack of statistical power in this subgroup analysis, this indicates that the strong association between reduced ETA and mortality is not due to a clear cardiac-specific cause of death such as arrhythmia-related sudden cardiac mortality. The mechanism by which low ETA values, which reflect blunted respiratory sinus arrhythmia, are linked to increased mortality remains elusive. On the one hand, impaired autonomic control of the heart may be detrimental to the heart itself, resulting in a higher probability of cardiac conditions such as myocardial infarction or heart failure. On the other hand, reduced respiratory sinus arrhythmia might just reflect the severity of co-morbidities, such as diabetes mellitus with autonomic neuropathy, which have their own detrimental effects on life expectancy. The lack of a clear association with cardiac-specific mortality in this study does not necessarily help us discriminate between these possibilities, given that cardiac co-morbidities may also be a limiting factor in primarily non-cardiac conditions such as cancer or infectious disease. In this regard, it is interesting to reflect on the common clinical finding that respiratory sinus arrhythmia is particularly pronounced in children and becomes physiologically blunted over time. Therefore—at the risk of oversimplification—it may be useful to view ETA as a surrogate of “biologic age” in autonomic cardiac control. Obviously, further mechanistic insight is required to understand the link between ETA and mortality.

Despite these limitations, the results of this study contribute important new insights into the role of autonomic regulation in aging and mortality. The ability of ETA to independently predict mortality, even when controlling for other risk factors, suggests that it is a valuable tool for assessing the cardiovascular health of elderly individuals. The combination of ETA with established clinical risk scores could improve the identification of high-risk individuals, enabling more personalized and targeted interventions to mitigate the risk of adverse outcomes.

In conclusion, we could demonstrate that ETA is a promising predictor of mortality risk in elderly individuals from the general population. By providing an additional layer of risk stratification, ETA complements traditional clinical measures and could help refine clinical decision-making in the management of older adults. Further studies are needed to confirm these findings, explore the mechanisms underlying the association between ETA and mortality, and determine the best ways to incorporate ETA into clinical practice for improved patient care.

## Figures and Tables

**Table 1:** Baseline characteristics of the study population.

Variable	Whole Cohort *n* = 1788	Survivors *n* = 1706	Non-Survivors *n* = 82	*p*
Age	72.00 [67.00, 76.00]	72.00 [67.00, 76.00]	75.00 [71.25, 78.00]	<0.001
Females	1032 (57.7%)	996 (58.4%)	36 (43.9%)	0.013
Hypertension	1376 (77.0%)	1311 (76.8%)	65 (79.3%)	0.708
Hyperlipidaemia	1045 (58.4%)	998 (58.5%)	47 (57.3%)	0.922
Diabetes mellitus	639 (35.7%)	601 (35.2%)	38 (46.3%)	0.053
Current smokers	514 (28.7%)	476 (27.9%)	38 (46.3%)	0.001
Manifest cardiovascular disease	438 (24.5)	400 (23.5%)	38 (46.3%)	<0.001
History of myocardial infarction	107 (6.0)	99 (5.8%)	8 (9.8%)	0.217
History of coronary artery disease	257 (14.4)	236 (13.8%)	21 (25.6%)	0.005
History of peripheral artery disease	153 (8.6)	134 (7.9%)	19 (23.5%)	<0.001
History of major stroke	118 (6.6)	106 (6.2%)	12 (14.6%)	0.006
Renal insufficiency	174 (9.7)	155 (9.1%)	19 (23.2%)	<0.001
Antihypertensive drugs	1355 (75.8)	1287 (75.4%)	68 (82.9%)	0.157
Statins	696 (38.9)	659 (38.6%)	37 (45.1%)	0.288
Modified Framingham score, %	14.40 [8.10–24.10]	14.15 [8.00, 23.17]	22.05 [13.23, 49.25]	<0.001
ETA (ms)	0.81 [0.10–1.81]	0.84 [0.12, 1.82]	0.41 [−0.55, 1.03]	<0.001
Follow-up (years)	4.0 [3.6–4.3]	-	-	-
All-cause mortality, *n*	82 (4.6)	-	-	-

Categorial variables are shown as absolute values (percentage) and continuous variables as median [interquartile range]. The *p* value is given for the comparison of survivors vs. non-survivors.

**Table 2:** Univariable and multivariable Cox analysis—dichotomized variables.

Variable	Univariable Model		Multivariable Model	
	HR	*p*	HR	*p*
ETA ≤ 0.19 ms	1.96 (1.26–3.03)	0.0026	1.81 (1.17–2.81)	0.008
Modified Framingham score ≥ 20%	2.53 (1.64–3.90)	<0.0001	2.40 (1.56–3.72)	<0.0001

HR: hazard ratio (95% confidence interval).

**Table 3:** Univariable and multivariable Cox analysis—continuous variables.

Variable	Univariable Model		Multivariable Model	
	HR	*p*	HR	*p*
ETA (per 1 ms)	0.93 (0.89–0.97)	0.0010	0.94 (0.90–0.99)	0.011
Modified Framingham score (per 1%)	1.04 (1.03–1.05)	<0.0001	1.04 (1.02–1.05)	<0.0001

HR: hazard ratio (95% confidence interval).

## Data Availability

Due to ethical restrictions, the data underlying this study cannot be made openly available. However, data are available from the corresponding author upon reasonable request.
